# Multimodal treatment of persistent postural–perceptual dizziness

**DOI:** 10.1002/brb3.1864

**Published:** 2020-09-28

**Authors:** Hubertus Axer, Sigrid Finn, Alexander Wassermann, Orlando Guntinas‐Lichius, Carsten M. Klingner, Otto W. Witte

**Affiliations:** ^1^ Center for Vertigo and Dizziness Department of Neurology Jena University Hospital Jena Germany; ^2^ Department of Otorhinolaryngology Jena University Hospital Jena Germany; ^3^ Biomagnetic Center Jena University Hospital Jena Germany

**Keywords:** cognitive–behavioral therapy, functional dizziness, multimodal treatment, persistent postural–perceptual dizziness, vestibular rehabilitation

## Abstract

**Background:**

Persistent postural–perceptual dizziness (PPPD) is a chronic disorder with fluctuating symptoms of dizziness, unsteadiness, or vertigo for at least three months. Its pathophysiological mechanisms give theoretical support for the use of multimodal treatment. However, there are different therapeutic programs and principles available, and their clinical effectiveness remains elusive.

**Methods:**

A database of patients who participated in a day care multimodal treatment program was analyzed regarding the therapeutic effects on PPPD. Vertigo Severity Scale (VSS) and Hospital Anxiety and Depression Scale (HADS) were assessed before and 6 months after therapy.

**Results:**

Of a total of 657 patients treated with a tertiary care multimodal treatment program, 46.4% met the criteria for PPPD. PPPD patients were younger than patients with somatic diagnoses and complained more distress due to dizziness. 63.6% completed the follow‐up questionnaire. All patients showed significant changes in VSS and HADS anxiety, but the PPPD patients generally showed a tendency to improve more than the patients with somatic diagnoses. The change in the autonomic–anxiety subscore of VSS only reached statistical significance when comparing PPPD with somatic diagnoses (*p* = .002).

**Conclusions:**

Therapeutic principles comprise cognitive–behavioral therapy, vestibular rehabilitation exercises, and serotonergic medication. However, large‐scale, randomized, controlled trials are still missing. Follow‐up observations after multimodal interdisciplinary therapy reveal an improvement in symptoms in most patients with chronic dizziness. The study was not designed to detect diagnosis‐specific effects, but patients with PPPD and patients with other vestibular disorders benefit from multimodal therapies.

## Graphical abstract

Therapeutic principles for persistent postural–perceptual dizziness (PPPD) comprise cognitive–behavioral therapy, vestibular rehabilitation exercises, and serotonergic medication. Follow‐up observations of patients subjected to multimodal interdisciplinary therapy reveal an improvement in symptoms in most patients with chronic dizziness.

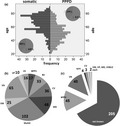

## 1 INTRODUCTION

Functional comorbidity is very common in patients with chronic dizziness (Staibano et al., 2019). Approximately 30%–50% of persistent dizziness cannot be fully explained by an identifiable medical illness (Schmid et al., 2011), and these patients mostly do not reveal any pathological results in technical diagnostics. Moreover, chronic dizziness and vertigo often are related to anxiety and depression, as well as to cognitive impairments (Lahmann et al., 2015).

In 2017, consensus criteria for persistent postural–perceptual dizziness (PPPD) have been defined by an expert panel (Staab et al., 2017). PPPD is a functional disorder that causes significant distress or functional impairment. It is characterized by symptoms of fluctuating dizziness, unsteadiness, and nonspinning vertigo on most days for at least three months (Popkirov et al., 2018). Upright posture, active, or passive motion without regard to direction or position, and exposure to moving visual stimuli or complex visual patterns may exaggerate symptoms. Mostly, the disorder is initially triggered by events that cause vertigo, unsteadiness, dizziness, or problems with balance. The symptoms cannot be better explained by another disease or disorder (Staab, 2020).

The definition of PPPD as a functional disorder is clearly separated from vestibular symptoms caused by a structural deficit of the vestibular system but also is distinctively separate from psychiatric causes (Staab et al., 2017). Functional disorders are considered as a change in the functioning of an organ unrelated to structural or cellular deficits (Dieterich & Staab, 2017). Consecutively, a change in functional connectivity in neuronal networks could recently be demonstrated in patients with PPPD (Li et al., 2020).

PPPD integrates earlier concepts of phobic postural vertigo, space motion discomfort, chronic subjective dizziness, and others (Dieterich & Staab, 2017). Therapeutic principles in PPPD comprise mainly cognitive–behavioral therapy (CBT), vestibular rehabilitation (VR), and the use of SSRI (selective serotonin reuptake inhibitors).

The principle of cognitive–behavioral therapy is based upon the premise that mental disorders and psychological distress are maintained by cognitive factors. Thus, CBT stimulates the patient to identify and to challenge the validity of maladaptive cognitions and therefore modify maladaptive behavioral patterns (Hofmann et al., 2012). CBT techniques applied in chronic dizziness comprise psychoeducation (information about dizziness), explanation and discussion of associations between assumptions (about dizziness), thoughts, moods and behaviors, behavioral experiments, exposure to feared stimuli, and attentional refocusing, coping strategies, and self‐observations (Edelman et al., 2012; Popkirov et al., 2018; Schmid et al., 2011). In addition, patients learn relaxation techniques.

Generally, the studies show a small but clinically relevant effect of CBT concerning dizziness (Edelman et al., 2012; Limburg et al., 2019; Schmid et al., 2011). However, in a one‐year follow‐up of 20 patients with phobic postural vertigo no treatment effect remained after CBT (Holmberg et al., 2007).

Vestibular rehabilitation (VR) is an exercise‐based group of approaches to train the system to overcome dizziness, vertigo, and balance disturbances. VR has especially been shown to improve symptoms after unilateral vestibular loss (McDonnell & Hillier, 2015). VR exercises are based upon different physiotherapeutic approaches (Kundakci et al., 2018; McDonnell & Hillier, 2015). Compensation is the ability of the brain to learn and, therefore, to change the functioning of central nervous networks. Substitution is a process that stimulates the use of intact sensory inputs (e.g., visual or somatosensory) in contrast to dysfunctional inputs (e.g., in the case of vestibular loss). Adaptation means that errors in visual–vestibular and balance systems can be corrected and readjusted. Habituation means that the system may reduce its responsiveness to motion stimuli in order to reduce the symptoms of dizziness.

Physical exercises and training comprise postural control exercises and gate stabilization, conditioning activities and occupational retraining, coordination training, and exercises for gaze stabilization. Recent studies (Nada et al., 2019; Thompson et al., 2015) found beneficial effects on patients with PPPD.

Selective serotonin reuptake inhibitors (SSRI) represent pharmacological options to modify anxiety and depression (Staab, 2016, 2020). Existing studies in chronic dizziness and vertigo are all open‐labeled and nonrandomized (Popkirov, et al., 2018) but show some beneficial effects (Horii et al., 2007; Staab & Ruckenstein, 2005; Staab et al., 2004). Sertraline and fluvoxamine were the most often used substances. However, the evidence level is relatively low.

As all these therapeutic effects are significant but rather limited, the use of a combination of these therapeutic principles suggests itself. Therefore, the aim of this study was to evaluate the effects of an interdisciplinary multimodal therapy program we use in our tertiary care specialized center for dizziness and vertigo. Here, we analyzed a database of 657 patients with chronic dizziness who participated in our day care multimodal treatment program with a focus on patients who met the diagnosis criteria for PPPD.

## 2 MATERIALS AND METHODS

We analyzed a database of patients with chronic dizziness who participated in a day care multimodal treatment program. The data were prospectively collected between June 2013 and March 2017 in the Center for Vertigo and Dizziness of Jena University Hospital. These data have already been analyzed to define age‐related characteristics of patients with chronic dizziness (Dietzek et al., 2018). The study was approved by the local ethics committee (ethics committee of the Friedrich‐Schiller‐University Jena, number 5426‐02/18). Written informed consent for study participation was obtained from all patients.

Multimodal and interdisciplinary day care treatment took place from Monday to Friday with an average of 7 hr of therapy per day. The therapeutic team consisted of a nurse, a neurologist, a psychologist, and a physiotherapist. The elements of the multimodal group therapy were specific physiotherapeutic training, CBT‐based psychoeducation and group therapy, training of Jacobson's muscle relaxation technique, and health education. The group sizes varied between 8 and 10 patients. In addition, every patient had an individual session with the psychologist for psychological assessment and counseling and an individual session with the neurologist for medical evaluation and treatment optimization as well. Table 1 shows the time schedule of the therapy week. Every patient had an outpatient consultation with a neurologist including a thorough diagnostic process before the patient was subjected to the therapy week.

**Table 1:** Time schedule of the therapy week

	Monday	Tuesday	Wednesday	Thursday	Friday
08:00–9:00	Address of welcome introduction	Health education	Individual session with psychologist or neurologist	Individual session with psychologist or neurologist	Individual session with psychologist or neurologist
9:00–10:30	Physiotherapy	Physiotherapy	Physiotherapy	Physiotherapy	Physiotherapy
10:30–12:00	CBT group	CBT group	CBT group	CBT group	Jacobson's progressive muscle relaxation
12:00–13:00	Lunch break	Lunch break	Lunch break	Lunch break	Lunch break
13:00–14:00	Individual session with psychologist or neurologist	Individual session with psychologist or neurologist	Individual session with psychologist or neurologist	Individual session with psychologist or neurologist	CBT group
14:00–15:00	Jacobson's progressive muscle relaxation	Jacobson's progressive muscle relaxation	Jacobson's progressive muscle relaxation	Jacobson's progressive muscle relaxation	Discharge
15:00–16:00	Individual session with psychologist or neurologist	Individual session with psychologist or neurologist	Individual session with psychologist or neurologist	Team visit	

All patients filled out a questionnaire before therapy begun. Age, gender, and medical diagnoses were collected. The patients were contacted via mail to fill out a second questionnaire six months after attendance of the day care therapy program.

The Vertigo Severity Scale (VSS) was used as assessment tool to quantify vertigo and dizziness symptoms (Yardley et al., 1992). The VSS comprises two subscales: vestibular–balance (VSS‐V) and autonomic–anxiety (VSS‐A) (Kondo et al., 2015). The Hospital Anxiety and Depression Scale (HADS) was used to screen for anxiety and depression (Andersson, 1993). In addition, the intensity of vertigo/dizziness and the distress due to vertigo/dizziness were quantified using a visual analog scale ranging from 0 to 10.

Statistics were performed using SPSS 21 (IBM Corp., IBM SPSS Statistics for Windows, Version 21.0.0). All data are reported as mean and standard deviation or as 95% confidence intervals. Paired and unpaired *t* tests were used appropriately for within‐ and between‐group comparisons. Change in scores in follow‐up assessments was represented as the difference between assessment scores before therapy and after 6 months follow‐up. As a beneficial effect is represented by a decline in score over time, differences become positive if symptoms decrease. Generally, a two‐sided significance level of *p* < .05% was assumed.

## 3 RESULTS

**Figure 1:**
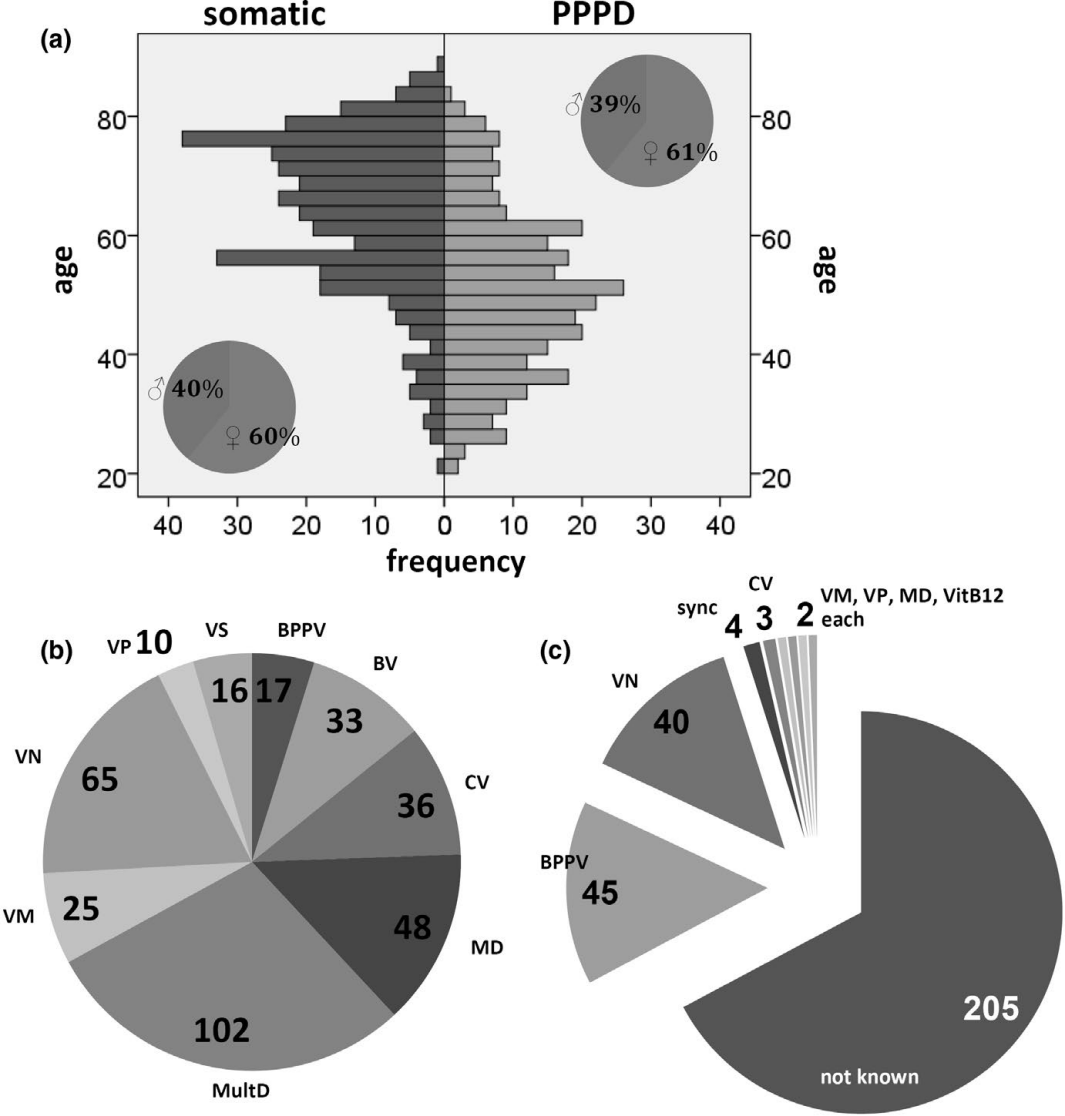
Clinical characteristics of the patients. (a) Age and gender. (b) Clinical diagnoses of the patients with somatic diagnoses. (c) Triggering and coexisting illnesses in PPPD patients. BPPV, benign paroxysmal positional vertigo; BV, bilateral vestibulopathy; CV, central vertigo; MD, Meniere's disease; MultD, multisensory deficit; sync, syncope; VitB12, vitamin B12 deficiency; VM, vestibular migraine; VN, vestibular neuritis; VP, vestibular paroxysmia; VS, vestibular schwannoma

A total of 657 patients (mean age 57.5 years, standard deviation 15.2, 60% female, and 40% male) were analyzed. Figure 1 summarizes the clinical characteristics of the patients.

Totally, 305 patients (46.4%) met the criteria for PPPD. In about one third of these patients, triggering and coexisting illnesses could be defined. Most of these were benign paroxysmal positional vertigo and vestibular neuritis (Figure 1c). The other 352 patients had primarily somatic diagnoses. Most of these diagnoses were multisensory deficit, vestibular neuritis, and Meniere's disease (Figure 1b). Table 2 shows the baseline characteristics of both patient groups. PPPD patients were younger than patients with somatic diagnoses and complained more distress due to dizziness. They had statistically significant higher scores in VVS, VSS‐A, and HADS anxiety.

**Table 2:** Baseline characteristics of both patient groups: age, visual analog scale of intensity of dizziness and distress by dizziness, Vertigo Severity Scale (VSS), and Hospital Anxiety and Depression Scale (HADS)

		Mean	*SD*	Significance
Age	PPPD	50.4	14.0	*p* < .001
	Other	63.9	13.2	
Intensity	PPPD	5.0	2.0	*p* = .609
	Other	5.1	2.1	
Distress	PPPD	6.0	2.2	*p* = .026
	Other	5.6	2.2	
VSS‐V	PPPD	12.0	8.7	*p* = .052
	Other	10.6	8.6	
VSS‐A	PPPD	15.6	10.7	*p* < .001
	Other	11.9	9.7	
VSS	PPPD	28.0	16.9	*p* < .001
	Other	22.6	15.2	
HADS anxiety	PPPD	7.8	4.2	*p* < .001
	Other	6.0	3.8	
HADS depression	PPPD	6.5	4.1	*p* = .054
	Other	5.8	3.9	

Four hundred and eighteen patients (63.6%) completed the follow‐up questionnaire six months after attendance of the therapy week with 183 PPPD patients and 235 patients with other somatic diagnoses. Table 3 shows the pre‐ and post‐treatment scores of both patient groups, as well as the mean differences between pre‐ and post‐treatment. Both groups showed significant changes in VSS, HADS anxiety, intensity, and distress. The PPPD patients generally showed a tendency toward a greater improvement compared to patients with somatic diagnoses. However, only the change in VSS‐A reached statistical significance (*p* = .002).

**Table 3:** Mean scores of visual analog scale of intensity of dizziness and distress by dizziness, Vertigo Severity Scale (VSS), and Hospital Anxiety and Depression Scale (HADS) of the 418 patients with pretreatment (pre) and post‐treatment follow‐up data six months after attendance of the therapy week (post). 183 patients met the diagnosis criteria of PPPD, and 235 patients had other somatic diagnoses

			Mean	*SD*	Mean of paired differences	*SD*	Significance
Intensity	PPPD	Pre	4.8	1.9	0.6	2.1	<0.001
		Post	4.3	1.5			
	Other	Pre	5.1	2.1	0.5	2.0	<0.001
		Post	4.5	2.1			
Distress	PPPD	Pre	5.7	2.1	1.1	2.6	<0.001
		Post	4.6	2.3			
	Other	Pre	5.5	2.1	0.6	2.5	<0.001
		Post	4.9	2.4			
VSS‐V	PPPD	Pre	11.7	8.4	2.5	9.1	<0.001
		Post	9.2	8.3			
	Other	Pre	10.7	8.3	2.4	10.8	0.001
		Post	8.3	8.6			
VSS‐A	PPPD	Pre	15.0	10.7	2.3	8.8	<0.001
		Post	12.7	9.8			
	Other	Pre	11.8	9.2	−0.2	7.9	0.672
		Post	12.0	9.0			
VSS	PPPD	Pre	26.7	16.7	4.8	15.3	<0.001
		Post	21.9	16.7			
	Other	Pre	22.5	14.5	2.2	15.1	0.026
		Post	20.3	15.0			
HADS anxiety	PPPD	Pre	7.7	4.0	0.6	3.5	0.016
		Post	7.0	4.2			
	Other	Pre	6.0	3.8	0.4	3.3	0.044
		Post	5.5	3.8			
HADS depression	PPPD	Pre	6.3	4.1	0.5	3.5	0.059
		Post	5.8	4.0			
	Other	Pre	5.8	3.8	0.1	2.8	0.784
		Post	5.7	3.6			

## 4 DISCUSSION

A major advantage of the PPPD concept is that it is a priori not necessarily connected to a specified underlying psychological process, and may be present alone or coexist with other conditions. In addition, a multifactorial pathophysiological concept is conjoined to PPPD (Seemungal & Passamonti, 2018).

Suggested mechanisms of PPPD initiation and sustainment are mainly based upon a maladaptive and dysfunctional process affecting systems for balance control and vestibular processing (Popkirov, et al., 2018). First of all, PPPD is triggered by an event of acute dizziness or vertigo, which could be not only a transient somatic dysfunction but also an acute psychological event such as a panic attack. A cycle of maladaptation (Popkirov, et al., 2018) leads to a persistence of symptoms, although the triggering event may already be dissolved. Predisposing factors may be originated in neurotic personality traits (Chiarella et al., 2016) or preexisting psychiatric disorders such as anxiety disorders or depression. Avoidance behavior, unfavorable thoughts, fears, and anxious self‐inspection are processes that may further sustain symptoms and lead to significant distress or functional impairment. The main goal of therapy is to discontinue this maladaptive cycle, and it is obvious that interventions with variable approaches may be suited to achieve this goal.

Therefore, treatment strategies of PPPD include patient education, vestibular rehabilitation, cognitive and behavioral therapies, and medication (Dieterich & Staab, 2017). Clinical studies analyzed different therapeutic principles (see Table 4), and all demonstrated beneficial effects. However, studies differ severely in regard to the applied therapeutic regimen, assessments and scores, and different times of follow‐up. In addition, they generally hamper from small sample sizes and partly fuzzy defined or just missing controls. Large‐scale, randomized, controlled trials of treatments for functional dizziness in PPPD are still missing. Nevertheless, existing therapy studies principally show promising effects mainly in CBT, VR, and the use of SSRI. It seems likely that a combination of all these therapeutic principles in a concerted multimodal interdisciplinary therapy concept may be beneficial for patients with chronic dizziness and vertigo, even if diagnose‐specific therapies already have been exhausted.

**Table 4:** Clinical studies

Publication	Diagnosis	*N*	Intervention	Control	Therapy	Assessments	Results
CBT—cognitive–behavioral therapy							
Limburg et al. (2019)	Functional vertigo and dizziness	72	Multimodal psychosomatic inpatient treatment	None	40‐day multimodal psychosomatic inpatient treatment	VHQ, VSS, PHQ‐15, SF‐36, PHQ‐15, BAI, BDI II	Medium effects for the change in vertigo‐related handicap and small effects for the change in somatization, mental quality of life, and depression
Edelman et al. (2012)	Chronic subjective dizziness	41 (*n* = 20 intervention, *n* = 21 controls)	3 weekly treatment sessions based on the CBT model of panic disorder	Wait‐list control	Psychoeducation, behavioral experiments, exposure to feared stimuli, and attentional refocusing	DHI, DASS‐21, Dizziness Symptoms Inventory, Safety Behaviors Inventory	Significant reductions in disability on DHI, reduced dizziness and related physical symptoms, and reduced avoidance and safety behaviors
Holmberg et al. (2006)	Phobic postural vertigo	39 (15 patients with self‐administered treatment, 16 patients with CBT completed the study)	10 sessions CBT + self‐administered treatment	Self‐administered treatment (education about the condition + self‐exposure by vestibular rehabilitation exercises)	CBT	DHI, VSS, HADS, VHQ	Larger effect in VHQ and HADS in the CBT group
Holmberg et al. (2007)	Phobic postural vertigo	20—one‐year follow‐up of the study above (Holmberg et al., 2006)					No significant treatment effects remained
Physical exercises/Vestibular rehabilitation therapy							
Nada et al. (2019)	PPPD	60 (*n* = 30 VRT, *n* = 30 VRT + placebo)	VRT + placebo	VRT	6 weeks, gait stabilization exercises + gaze stabilization exercises	DHI	Significant decrease in functional, physical, and total scores on the DHI in both groups after VRT
Thompson et al. (2015)	PPPD	26 (12 with PPPD alone, 8 with PPPD plus vestibular migraine, and 6 with PPPD and vestibular deficits)	Vestibular and balance rehabilitation therapy (VBRT)	None	Balance exercises, visual habituation for motion and patterns, habituation for head and body motion, Habituation for complex environment, diaphragmatic breathing, aerobic exercise, and neck stretches	DHI, HADS	22 of 26 participants found physical therapy consultation helpful. 14 found VBRT exercises beneficial
Medication							
Horii et al. (2007)	Dizziness without pathological results	19 patients with neuro‐otologic diseases, 22 patients without abnormal findings in standard vestibular tests	SSRI	Patients with neuro‐otologic diseases	Fluvoxamine	HADS, stress hormones (vasopressin and cortisol)	Fluvoxamine decreased subjective handicaps of both groups
Staab and Ruckenstein (2005)	Chronic subjective dizziness	88 (28 with otogenic pattern, 31 with psychogenic pattern, and 29 with interactive pattern)	SSRI	3 groups: otogenic, psychogenic, and interactive	Sertraline hydrochloride, fluoxetine hydrochloride, paroxetine, citalopram hydrobromide, or escitalopram oxalate	CGI‐I	Patients with the otogenic and psychogenic patterns had a more complete response than did patients in the interactive group (*p* 0.01).
Staab et al. (2004)	Chronic subjective dizziness	24	Sertraline	None	16‐week open‐dose sertraline therapy	DHI, BSI‐53	Sertraline significantly reduced scores on all three DHI subscales and the BSI‐53. positive response rate of 55% for the entire cohort and of 73% for those who completed treatment
Others							
Eren et al. (2018)	PPPD	nVNS group (*n* = 10) vs. SOC (*n* = 9), former SOC group also received nVNS for additional for 4 weeks, merged group for the pooled analysis consisted of 16 patients	Noninvasive vagus nerve stimulation (nVNS)	SOC: detailed psychoeducation of the pathophysiology of their PPPD (minimally 30‐min) and reinforcing physical activity and relaxation exercises	nVNS	EQ‐5D‐3L, HADS	Patients in the vagus nerve stimulation period had a significant improvement in the quality of life and in the depression scores
Combined therapy							
Yu et al. (2018)	PPPD	91 (45 controls, 46 experiment group)	CBT + sertraline	Sertraline	CBT	DHI, HARS, HDRS	Both sertraline as monotherapy and sertraline + CBT could significantly reduce the average DHI scores, HDRS scores, and HARS scores. Sertraline + CBT could yield significantly lower average DHI score, HDRS score, and HARS score
Andersson et al. (2006)	Chronic dizziness	21 (*n* = 14 intervention, *n* = 15 controls)	Combined cognitive–behavioral/vestibular rehabilitation (VR) program 5 sessions and one individual telephone call over a period of 7 weeks	Wait‐list control	Psychoeducation, vestibular exercises, relaxation, and cognitive intervention	DHI, VSS, CEA, STAI‐T, BDI, PSS, behavioral measures, diary registrations	On the VSS, a significant interaction effect was found, with the treatment group improving and the control group remaining stable

Abbreviations: BAI, Beck Anxiety Inventory; BDI, Beck Depression Inventory; BSI‐53, Brief Symptom Inventory‐53; CBT, cognitive–behavioral therapy; CEA, Confidence in Everyday Activities questionnaire; CGI‐I, Clinical Global Impressions–Improvement Scale; DASS‐21, Depression, Anxiety, and Stress Scales 21; DHI, Dizziness Handicap Inventory; EQ‐5D‐3L, European Quality of Life 5 Dimensions 3 Level Version; HADS, Hospital Anxiety and Depression Scale; HARS, Hamilton Anxiety Rating Scale; HDRS, Hamilton Depression Rating Scale; PHQ‐15, Patient Health Questionnaire‐15; PSS, Perceived Stress Scale; SF‐36, Short Form Health Survey; SOC, standard of care; STAI‐T, Spielberger State–Trait Anxiety Inventory—Trait form; VBRT, vestibular and balance rehabilitation therapy; VHQ, Vertigo Handicap Questionnaire; VRT, vestibular rehabilitation therapy; VSS, Vertigo Severity Scale.

In recent years, specialized tertiary care centers were developed in Germany providing multimodal interdisciplinary therapy programs for the treatment of chronic dizziness and vertigo. Positive long‐term effects have been found following a thorough diagnostic process and specific treatment, which could be demonstrated to be persistent over a period of 2 years (Obermann et al., 2015). Patients with benign paroxysmal positional vertigo and phobic postural vertigo showed the largest changes in Dizziness Handicap Inventory (DHI).

The multimodal interdisciplinary therapy program, as provided in the Center for Vertigo and Dizziness of Jena University Hospital, is a 5 day care outpatient therapy program for patients with chronic dizziness. The aim of the therapy is explicitly not to completely eliminate the symptoms after one week of therapy. In contrast, the patients are motivated to learn various techniques and methods to alleviate symptoms, which should be regularly practiced in follow‐up.

**Figure 2:**
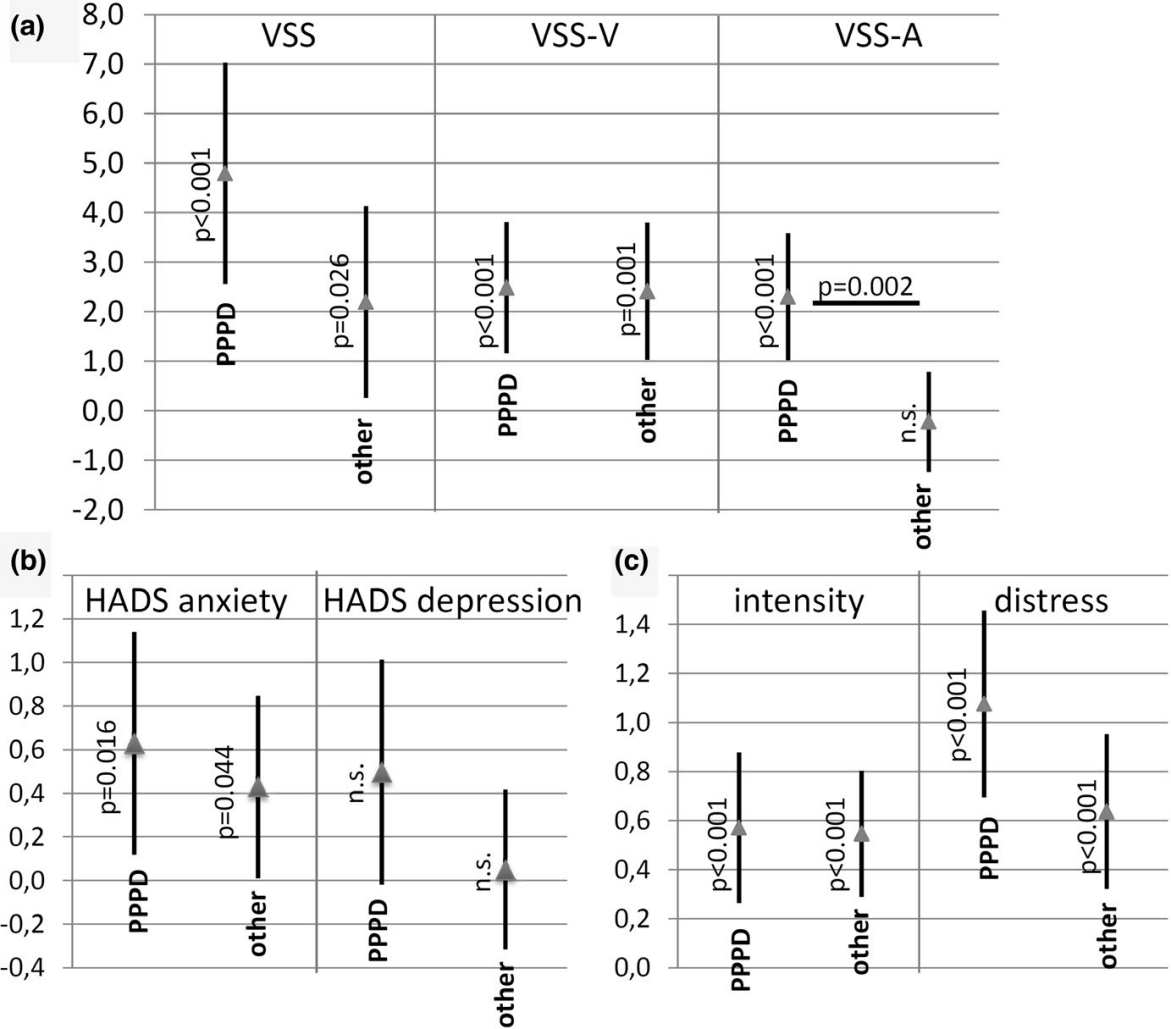
Change in scores before and 6 months after therapy week. (a) VSS. (b) HADS, (c) visual analog scale of intensity of dizziness and distress due to dizziness. The change is shown as 95% confidence interval and mean of differences. Note that these differences are calculated from pretreatment scores minus post‐treatment scores. A clinical improvement is represented by a positive value of the difference

Our data demonstrate that the majority of patients show beneficial effects in all VSS scores and in HADS anxiety scores at the follow‐up 6 months after therapy. Figure 2 shows the change in (paired) scores after therapy. For the patients who fulfilled the criteria of PPPD, the change in the autonomic–anxiety subscore of the VSS (VSS‐A) was larger than in the patients with other somatic diagnoses (*p* = .002).

It has to be kept in mind that—although patient numbers are high—the analysis is a simple follow‐up observation and not a randomized, controlled trial. In addition, this study encompasses a follow‐up time of 6 months. It would also be very interesting to analyze outcomes after a longer time interval, especially as some of other therapy studies have not been able to show a relevant long time effect (Holmberg et al., 2007). Therefore, we plan to perform a second survey after a longer time course of follow‐up.

Both patient groups received the same therapy program. Thus, the therapy program was basically transdiagnostic and therefore may provide nonspecific benefits for patients with many vestibular disorders. The study protocol was not designed to detect diagnosis‐specific effects. However, the readjustment of a dysfunctioning vestibular system can be of benefit in functional‐, somatic‐, and psychiatric‐dominated conditions as well—in particular, a considerable overlap between these entities exists (Staab et al., 2017).

Moreover, the relatively high portion of PPPD patients (46.4%), who took part in the therapy program, is remarkable. That may be due to the fact that especially those patients with functional vestibular disturbances may be regarded as to profit from multimodal therapy. In addition, suited therapeutic offers are quite limited for patients with chronic dizziness in the outpatient setting.

## 5 CONCLUSION

Therapeutic principles to treat PPPD comprise cognitive–behavioral therapy, vestibular rehabilitation exercises, and medication (i.e., SSRI). Although therapy studies for PPPD and PPPD‐like disturbances to date have been promising, large‐scale, randomized, controlled trials are still missing. Follow‐up observations of multimodal, interdisciplinary therapy programs that represent a concerted combination of different principles of therapy reveal an improvement in the symptoms of most patients with chronic dizziness. Thus, patients with PPPD and patients with other vestibular disorders benefit from these therapies. In our opinion, the concept of PPPD is most helpful for patients with chronic dizziness to provide a conceptual backbone for the planning and creation of multidisciplinary therapy programs.

## CONFLICT OF INTEREST

The authors declare no financial or other conflicts of interest.

## AUTHOR CONTRIBUTIONS

HA, OWW, and AGL conceptualized and designed the study. SF, AW, and HA collected the data. SF, HA, and CMK analyzed the data. HA and CMK drafted the manuscript. All authors critically reviewed and finally approved the manuscript.

### Peer Review

The peer review history for this article is available at https://publons.com/publon/10.1002/brb3.1864.

## Data Availability

The data that support the findings of this study are available from the corresponding author upon reasonable request.
